# Vitamin D insufficiency is high in Malaysia: A systematic review and meta-analysis of studies on vitamin D status in Malaysia

**DOI:** 10.3389/fnut.2022.1050745

**Published:** 2022-11-18

**Authors:** Shamin Mohd Saffian, Nor Aini Jamil, Nor Asyikin Mohd Tahir, Ernieda Hatah

**Affiliations:** ^1^Centre for Quality Management of Medicines, Faculty of Pharmacy, Universiti Kebangsaan Malaysia, Kuala Lumpur, Malaysia; ^2^Centre for Community Health Studies, Faculty of Health Sciences, Universiti Kebangsaan Malaysia, Kuala Lumpur, Malaysia

**Keywords:** Malaysia, vitamin D, 25-hydroxyvitamin D, prevalence, cholecalciferol

## Abstract

**Purpose:**

To estimate the vitamin D status of participants residing in Malaysia.

**Methods:**

PubMed, Scopus, Web of Science, and MyJurnal were searched up to June 2022 without language restrictions. Studies that reported the 25-hydroxyvitamin D [25(OH)D] concentrations and defined their cut-off for deficiency or insufficiency from healthy participants residing in Malaysia were included. The random effects model was used to pool vitamin D status using established cut-offs of <30, <50, and <75 nmol/L according to age group.

**Results:**

From 299 studies screened, 32 studies were included in the meta-analysis. The pooled proportion for <30 nmol/L was 21% (95% CI 9–36, *n* = 2,438 from 10 studies), while the pooled proportion <50 nmol/L was 64% (95% CI 56–72, *n* = 13,977 from 30 studies), and <75 nmol/L was 85% (95% CI 61–100, *n* = 1,376 from five studies). Heterogeneity was high (I^2^ ranged from 98–99%). Higher proportions of vitamin D insufficiency (defined as <50 nmol/L) were found in participants living in the urban areas (compared to rural areas), in females (compared to males), and in Malays and Malaysian Indians (compared to Malaysian Chinese) ethnicities.

**Conclusion:**

More than half of Malaysians have insufficient vitamin D levels, despite being a country that is close to the equator. We strongly urge prompt public health measures to improve the vitamin D status in Malaysia.

**Systematic review registration:**

[https://www.crd.york.ac.uk/prospero/], identifier [CRD42021260259].

## Introduction

Vitamin D is well-recognized as a hormone that plays an essential role in maintaining adequate serum calcium and phosphate concentrations and bone health. The importance of vitamin D in maintaining a sound immune system has also been well-discussed (1). Following the coronavirus disease-2019 (COVID-19) pandemic, there has been a renewed interest in understanding the role of vitamin D in managing COVID-19 infection (2). Several systematic reviews have demonstrated that vitamin D levels are associated with the severity of COVID-19 conditions (3–11). This is echoed by several clinical trials showing positive outcomes of supplementing vitamin D in COVID-19 infection, especially in deficient patients (8, 12–15). Although the exact benefit of vitamin D in COVID-19 management remains debatable (16–19), the importance of maintaining adequate vitamin D levels for general health is not disputed.

There is no consensus on the optimal vitamin D concentration in the body [measured as 25-hydroxyvitamin D; 25(OH)D]. It is generally agreed that concentrations <30 nmol/L (or 12 ng/ml) should be avoided in all age groups (20), but the definition of deficiency and insufficiency varies between guidelines. The Endocrine Society Clinical Practice Guidelines 2011 defines vitamin D deficiency as 25(OH)D concentrations <50 nmol/L (or <20 ng/ml), while 50–75 nmol/L (or 20–30 ng/ml) is defined as insufficient (21). The Endocrine Society further recommends levels above 75 nmol/L to reduce the risk of infectious diseases and to obtain other non-calcemic benefits of vitamin D. On the other hand, the Institute of Medicine (IOM) defines vitamin D deficiency as <30 nmol/L, while 30–50 nmol/L is insufficient (22). The IOM have used bone health as the main basis for their recommendations. Misra et al. (23), define vitamin D deficiency as ≤37.5 nmol/L and insufficiency as 37.5–50 nmol/L (23). Although there is no consensus on the terminology, a cut-off of <50 nmol/L is commonly used between guidelines. At this concentration, there are compensatory mechanisms activated to maintain calcium homeostasis, which will affect bone and muscle health (24).

Malaysia is a Southeast Asian country close to the equator, with latitudes ranging from 1.2°N to 6.8°N. Malaysia consists of two regions: Peninsular Malaysia, which houses approximately 80% of the Malaysian population, and Malaysian Borneo. It has an equatorial climate with relatively stable temperatures all year that are hot, sunny, and humid with an average daily temperature that ranges between 21 and 32°C. Seasonal climate variability is closely tied to the drier Southwest Monsoon, which occurs from April to September, and the wetter Northeast Monsoon, from October to March (25). In theory, during periods of heavy rainfall, more of the population may be confined indoors, limiting their sun exposure during the day. However, in a recent study, Aris et al. (26) reported that while there was a significant decrease in vitamin D concentrations during the monsoon season for some subgroup of individuals, there was no significant difference in the vitamin D status between monsoon and non-monsoon season (26). Approximately 69.8% of the 29.9 million Malaysian population is Malay and Bumiputera, 22.4% is Chinese (i.e., Malaysian Chinese), 6.8% is Indian (i.e., Malaysian Indian), and the remainder is other ethnicities (27). Malaysian Chinese generally have fair skin tone, whereas, Malays’ skin tone may vary from light to tanned, and most Malaysian-Indians have darker skin tone (28). As vitamin D is synthesized in the skin following sunlight exposure and is highly correlated with skin color, the Malay and Malaysian Indian populations are at higher risk of lower vitamin D status.

A recent review article has highlighted several studies have investigated the vitamin D status of subpopulations in Malaysia (29). Most of the results have indicated that vitamin D status is low in Malaysia. However, the prevalence of vitamin D status has not been pooled across studies. Therefore, the primary aim of this research is to combine the data from studies that have been conducted on the vitamin D status in populations living in Malaysia.

## Methods

This systematic review was conducted following the PRISMA guidelines (37) and was registered in PROSPERO (CRD42021260259).

### Identifying published studies

We searched through PubMed, Scopus, Web of Science, and MyJurnal (a Malaysian Journal database) from the inception of each database until June 2022. A systematic search was conducted to identify all studies that reported vitamin D status in Malaysian residents. The search terms included (Vitamin D OR its synonyms) AND (Malaysia OR its synonyms). The full record of the search strategy is presented in Supplementary material 1. We did not use any language or other search limits. Key review publications were also identified and searched for further relevant studies. In addition, the reference lists from the identified studies were also examined for potentially relevant studies.

### Inclusion and exclusion criteria

For a study to be included in the systematic review, there were two inclusion criteria: (i) the study measured serum 25(OH)D levels in healthy Malaysians, and (ii) it was non-interventional study and conducted either as a cross-sectional, case-control, or longitudinal study design. For longitudinal studies, baseline vitamin D levels must be provided. Studies were excluded if they were: (i) case reports or case series, or (ii) meeting abstracts or unpublished materials, (iii) articles that were not written in English, (iv) review articles and meta-analyses.

The meta-analysis considered only data from healthy population subgroups for case-control studies. We excluded studies where the lifestyle differed significantly from Malaysia’s general population from the meta-analysis. If a subsequent research included data from a previous cohort of individuals, the study with the larger sample size would be included in the meta-analysis.

Three investigators (NAJ, SMS, NAMT) developed the search strategy. Then, SMS and NAMT performed the database search and independently screened the retrieved articles based on the titles and abstracts. All four authors assessed the full-text articles and selected studies based on the inclusion and exclusion criteria. Any disagreements in the study selection were resolved by consensus. All data were extracted from the selected studies using a standardized extraction form, including information on the study location, data collection period, population demographics (ethnicity, sex, age), assay method, vitamin D status (including cut-off definitions), and vitamin D concentrations.

### Critical appraisal of studies included

After considering various quality assessment tools for prevalence studies (38), the Joanna Briggs Institute Prevalence Critical Appraisal Tool, 2014 (39, 40) was used for quality assessment. Three investigators (EMH, NAJ, and SMS) critically appraised and rated the studies included in the meta-analysis using the tool. The tool consists of nine questions with four standard answer options (*yes/no/unclear*/*not applicable*). Each rater rated independently; however, discussions were carried out to ensure that discrepancies were discussed and agreed upon through consensus.

### Statistical analysis

For the overall data, we performed a meta-analysis using three predetermined vitamin D cut-off values (<30, <50, and <75 nmol/L) sub-grouped by age category and a meta-analysis of mean 25(OH)D levels. The random-effects model was used to pool the proportion and mean values given an *a priori* assumption of heterogeneity between prevalence studies (41). If the mean and standard deviation were not provided, they were estimated from the median, interquartile range, range, and sample size, assuming that the data were not significantly skewed (42). For all meta-analyses of specific subgroup proportions (e.g., gender, ethnicity), a cut-off of <50 nmol/L was used, in view that this cut-off is present in almost all of the studies. We examined the presence of heterogeneity using the Q statistic with *P* < 0.05 indicating significant heterogeneity exists. The heterogeneity between studies was then quantified using the I^2^ statistics with values of <25, <50, and <75% indicating low, moderate, and high, and the χ2 test with *P* < 0.05 to denote significance (43). All analyses were performed using MetaXL, version 5.3 (EpiGear International, Sunrise Beach, QLD, Australia). A sensitivity analysis is, by default, reported by MetaXL by excluding one study at a time and recalculating the pooled effect sizes and the associated heterogeneity statistics. The funnel plot was not used as it is inaccurate to assess publication bias for prevalence studies (44). The overall mean vitamin D levels and the proportion of vitamin D deficiency/insufficiency were also summarized by study location.

## Results

### Literature search results and characteristics of the eligible studies

**FIGURE 1:**
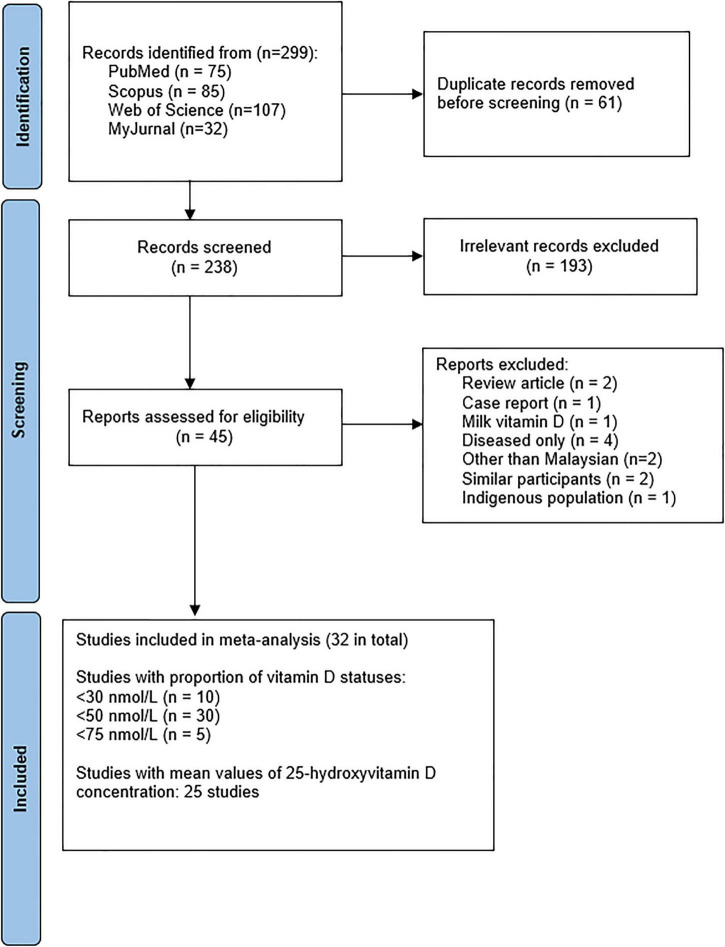
Flow-chart of study selection.

From the 299 articles identified, 45 full-text articles were assessed, and 32 studies were included in the systematic review and meta-analysis (see Figure 1). Table 1 shows the characteristics of the studies included in the systematic review. Two studies were published before 2010 (45, 46). Twenty-five studies were mainly from participants in Malaysia’s capital city (Kuala Lumpur and its surrounding areas). Four studies were excluded as they only presented data on specific patients (47–50) and did not include healthy controls. One study on indigenous people (51) was excluded because their lifestyles differed significantly from the rest of the population. Two studies were excluded from the meta-analysis because it has part of the same individuals from a previous research (52, 53). The summary the of study quality assessment is presented in Supplementary material 2. The included studies measured 25(OH)D levels using immunoassays, except for four studies that used chromatographic methods (54–57).

**TABLE 1:** Characteristics of studies included in the systematic review.

References	State	Period data was collected	Total sample size (*n*), population	*n*, Ethnicity	Male (%)	Vitamin D measurement method	Number of participants, vitamin D classification [25 (OH)D concentrations are in nmol/L]
Rahman et al. (45)	Kuala Lumpur	Not reported	274, postmenopausal women	101, Malay 173, Chinese	0	Radio-immunoassay	2, <25 deficient 93, 25–50 insufficient 179, 50–100 hypovitaminosis
Green et al. (46)	Kuala Lumpur	January and March 2005	378, non-pregnant women	133 Malay 123 Chinese 122 Indian	0	Radio-immunoassay	113, <17.5 deficient 227, <50 insufficient
Khor et al. (81)	Kuala Lumpur	2008	402, from five primary school children	51.2% Malay 42.8% Chinese 6% Indian and others	44.8	CLIA	142, <37.5 deficient 149, <37.5–50 insufficient
Moy and Bulgiba (71)	Kuala Lumpur	May to July 2010	380, Malay employees	100% Malay	41.6	CLIA	258, <50 insufficient
Hawa et al. (82)	Kelantan	Not reported	150, (51 pre-menopausal and 99 post-menopausal)	100% Malay	0	ELISA	1, <25 deficient 124, 25–49.8 insufficient 25, 50–100 hypovitaminosis
Nurbazlin et al. (83)	Kuala Lumpur and Negeri Sembilan	Aug 2010 to July 2011	400, women (293 urban, 107 rural)	267, Malay 67, Chinese 66, Indian	0	ECLIA	48, <30 deficient 74, 30–50 insufficient 278, ≥50 sufficient
Poh et al. (84)	Six regions in Malaysia (northern, central, southern, east coast, Sabah & Sarawak)	May 2010 to October 2011	2,936, children 4–12 years old	Not reported	49.7	CLIA	1,395, <50 insufficient
Chin et al. (85)	Klang Valley	September 2009 to September 2011	383, men	Malay (39.2%), Chinese (60.8%)	100	ELISA	2, <30 deficient 85, 30–50 insufficient
Jan Mohamed et al. (56)	Kelantan	April 2010 to December 2012	102, pregnant women	100% Malay	0	HPLC	4, <25 severe deficient 57, 25–50 mild deficient 35, 50–75 insufficient
Al-Sadat et al. (86)	Perak, Selangor and Kuala Lumpur	March 2012 to May 2012	1,361 school students	Malay 1091 (80.2) Chinese 105 (7.7) Indian 105 (7.7) Others 41 (3)	38.6	ECLIA	20, <12.5 severe deficient 1,053, 12.5–37.5 deficient 187, 37.5–50 insufficient
Shafinaz and Moy (87)	Kuala Lumpur	February to May 2013	858 teachers from 30 schools	76.9% Malay 16.4% Chinese 8.3% Indian 0.4% Others	9.1	ECLIA	578, <50 deficient
Bukhary et al. (75)	Selangor	January to April 2014	396, pregnant women	77.5% Malay 11.2% Chinese 8.8% Indian 2.5% Others	0	ECLIA	174, <24.99 184, 25–49.99 deficient 33, 50–74.99
Lee et al. (55)	Kuala Lumpur	August 2013 to August 2015	575 women completed 37 weeks of pregnancy (term)	73.4% Malay 18.4% Chinese 4.9% Indian 3.3% others	0	UPLC	412, <50 deficient 121, 50–75 insufficient
Moy et al. (88)	Kuala Lumpur	March to October 2013	770, female teachers	76.6% Malay 15% Chinese 8.4% Indian	0	ECLIA	557, <50 deficient
Rahmadhani et al. (70)	Kuala Lumpur	January 2012 to July 2012.	941 boys and girls from 23 schools	75% Malay 13% Chinese 10% Indian 2% Others	28	ECLIA	305, <37.5 deficient 166, 37.5–50 insufficient
Ralph et al. (54)	Kota Kinabalu, Sabah	Ethics approved in 2010/1	92, controls for cases (tuberculosis)	Not reported	35.8	LCMS	23, <50 deficient
Ariffin et al. (78)	Kuala Lumpur	March to August 2017	57, pregnant women	86% Malay 14% non-Malay	0	ELISA	15, <25 severe deficient 37, 25-49.9 mild deficient
Mat et al. (60)	Kuala Lumpur	November 2013 to October 2015	1,011, elderly	31% Malay 36.3% Chinese 32.7 Indian	43	CLIA	409, <50 deficient
Quah et al. (89)	Selangor, Perak and Kuala Lumpur	1 April 2014 to 30 June 2014	1,016 students (14–15-year-olds) from 15 urban and rural schools	Not reported	38	CLIA	338, ≤50 678, >50
Jamil et al. (74)	Kuala Lumpur	March to July 2019	147, Malay office workers	100% Malay	0	Enzymatic immunoassay	133, <50 insufficient
Lee et al. (90)	Johor Bahru	1st December 2016 and 31st May 2017	65, controls for cases (atopic dermatitis)	67.7% Malay 16.9% Chinese 5% Indian 5% Others	52.3	ECLIA	19, <50 deficient 22, 50–75 insufficient
Abd Aziz et al. (91)	Kuala Lumpur	Not stated, ethics approved in 2018	60, women	87% Malay 7% Chinese 5% Indian	0	ELISA	6, <30 deficiency 34, 30–50 insufficiency
Arumugam et al. (92)	Kuala Lumpur	June 2014 to February 2015	38, controls for cases (atopic dermatitis)	Not reported	36.8	ECLIA	1, <30 deficient 22, 30–50 insufficient
Ismail et al. (93)	Kuala Lumpur	Not reported	78, pregnant women	77% Malay 15.4% Chinese 5.1% Indian 2.5% Others	0	ECLIA	27, <30 deficient 36, 30–50 inadequate
Leiu et al. (94)	Kuala Lumpur and Selangor.	Not reported	214, post-menopausal Chinese women	100% Chinese	0	CLIA	71, <30 deficient 106, 30–50 insufficient
Ismail et al. (95)	Kelantan	August 2017 to October 2017	126, healthy volunteers	100% Malay	43.7	CLIA	92, <30 deficient 12, 30–50 insufficient
Woon et al. (58)	Kuala Lumpur and Selangor	November 2016 and January 2018	535, late pregnancy	Not reported	0	CLIA	227, <30 deficient
Aris et al. (26)	Kelantan	May to June 2012	119, indoor workers, 119, outdoor workers	100% Malay	31 (indoor) 93.3 (outdoor)	ECLIA	64, <50 deficient 46, 50–75 insufficient
Hussain and Elnajeh (59)	Kelantan	Not stated, ethics approved in 2012	361, adolescents from 10, schools	85% Malay 15% Chinese	37.1	ECLIA	59, <30 deficiency
Chee et al. (96)	Kuala Lumpur	August 2017 to August 2019	243, 9- to 11-year-olds	90.5% Malay 9.5% Indian	52.3	LC–MS/MS	46, <30 deficient 123, <50 inadequate
Mustapa Kamal Basha et al. (57)	Kuala Lumpur	November 2017 to March 2019	179, longitudinal study of pregnant women	78% Malay 13.6% Chinese 6.8% Indian 1.7% others	0	HPLC	161, <50 deficient
Razip et al. (97)	Selangor	Not reported	50, controls for adult diabetes patients aged 30 to 65	90% Malay 2% Chinese 8% Indian	56	HPLC	41, <50 deficient 8, 50–200 Optimal 1, >200 Toxic

HPLC, high-performance liquid chromatography; LC-MS/MS, liquid chromatography with tandem mass spectrometry; ECLIA, electrochemiluminescence immunoassay; ELISA, enzyme-linked immunosorbent assay; CLIA, chemiluminescent immunoassay.

### Meta-analyses

**FIGURE 2:**
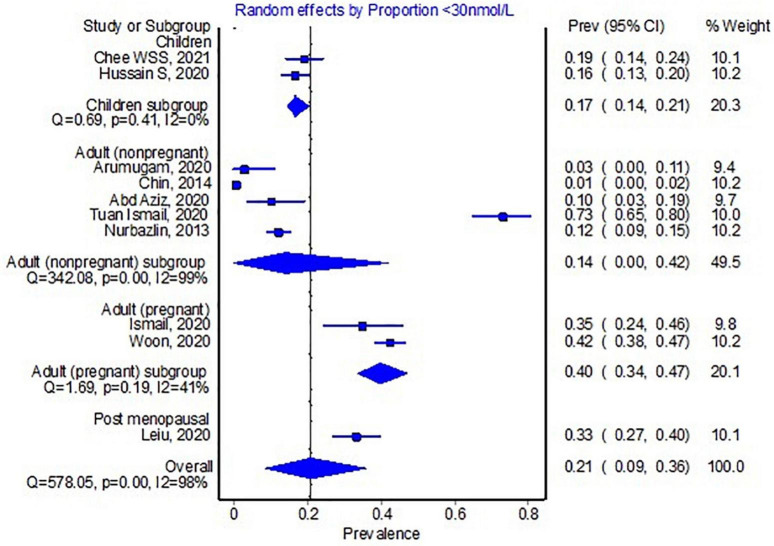
Pooled proportion of the <30 nmol/L 25-hydroxyvitamin D cut-off.

For the <30 nmol/L cut-off, ten studies with a total of 2,438 participants were included. The pooled proportion for this cut-off was 21% (95% CI 9–36%, see Figure 2). Pregnant women had a higher proportion of participants with 25(OH)D levels <30 nmol/L (*n* = 613, 40%).

**FIGURE 3:**
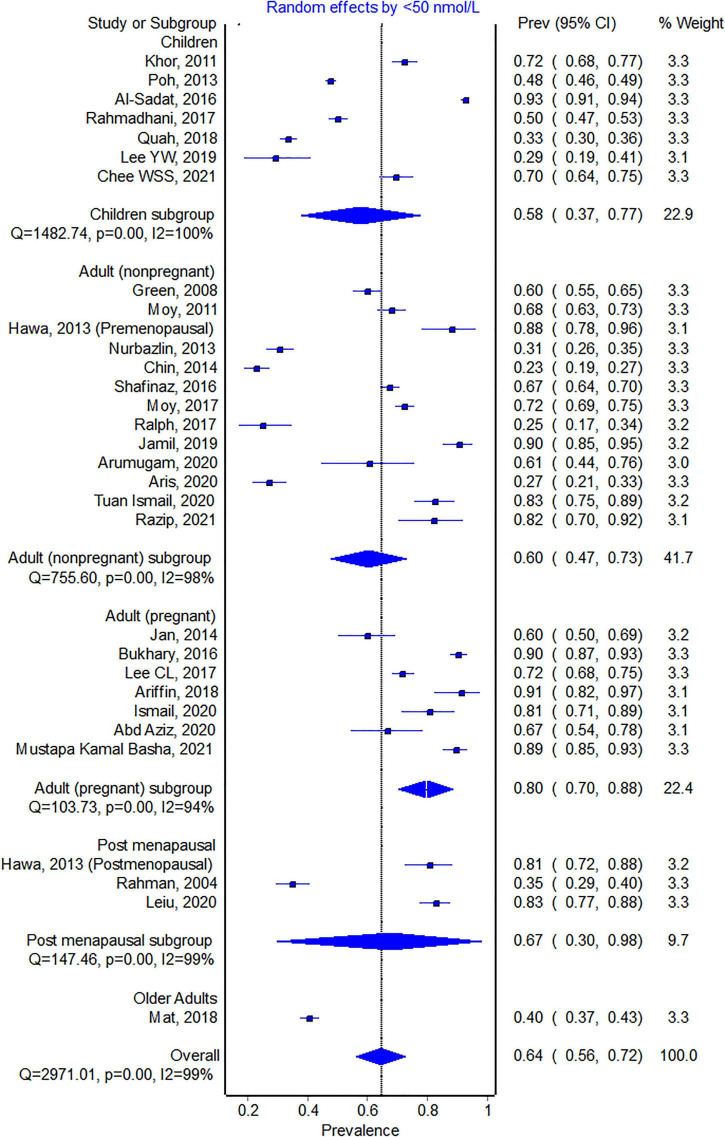
Pooled proportion of the <50 nmol/L 25-hydroxyvitamin D cut-off.

All studies eligible for the meta-analysis, except for two studies (58, 59), had a <50 nmol/L cut-off. The pooled proportion for <50 nmol/L from three studies with data from 13,977 individuals was 64.5% (95% CI 56.1–72.5, see Figure 3). Again, pregnant women represent the highest proportion with vitamin D levels of <50 nmol/L. The pooled proportion for each subgroup of participants was above 50% except for one study (60). Mat et al. (60) conducted a study on adults above 55 years old, and 203 out of the 1,011 participants were taking vitamin D supplements, which could explain the lower proportion of individuals with levels <50 nmol/L.

**FIGURE 4:**
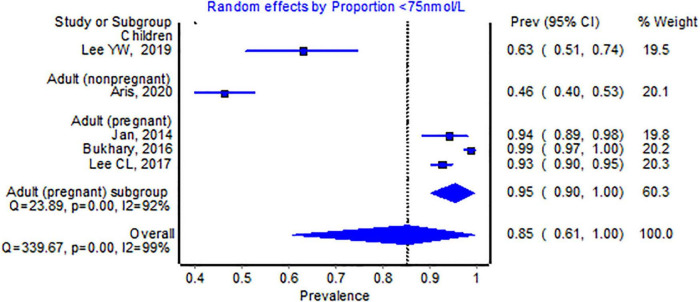
Pooled proportion of the <75 nmol/L 25-hydroxyvitamin D cut-off.

Only five studies with 1,376 participants reported a cut-off of <75 nmol/L, and the pooled proportion was 85% (95% CI 61–100, see Figure 4). All pooled proportions, including most subgroups, were considered high, indicating substantial heterogeneity (>99%).

**FIGURE 5:**
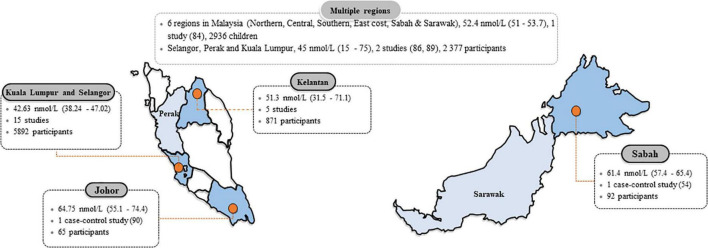
Mean 25-hydroxyvitamin D [25(OH)D] concentrations in Malaysia according to states. Data are the mean (95% confidence interval) of 25(OH)D concentrations reported in studies done in each state. The 25(OH)D mean values were pooled if there were more than one study from a form and were computed only from studies that stated the mean (SD), or median and interquartile range or range.

The pooled mean (95% CI) according to the location of the study is reported in Figure 5. Not surprisingly, the urban population had a higher proportion of vitamin D insufficiency (66.8%, 95% CI 57.8–75.3, *n* = 10,893) compared to the rural population (45.6%, 95% CI 21–71.1, *n* = 3,487), respectively (see Supplementary materials 3, 4). Chua et al. (51) conducted a study on the indigenous population, considered a significant outlier and thus excluded from the meta-analysis. However, Chua et al. reported that only 1.4% (out of 555 indigenous people) had 25(OH)D levels <50 nmol/L, and 26.3% had levels <75 nmol/L.

Vitamin D insufficiency is also more common in females (76%, 95% CI 65–86, *n* = 6,264, see Supplementary material 5) compared to males (46%, 95% CI 25.6–67%, *n* = 3,664, see Supplementary material 6). In terms of ethnicity, Malays (77%, 95% CI 65–87; *n* = 4,928, see Supplementary material 7) and Indians (77%, 95% CI 57–92; *n* = 768, see Supplementary material 8) had higher proportions of vitamin D insufficiency than the Chinese population (34.5%, 95%CI 17–54; *n* = 1,353, Supplementary material 9). One study examined the relationship between monsoon seasonality and vitamin D status and found no significant association (26).

## Discussion

This study found that vitamin D deficiency and insufficiency, as defined by three cut-offs, is common in Malaysia. Precisely, over half of the population is estimated to have 25(OH)D levels <50 nmol/L (see Figure 3). Only two studies (45, 46) were conducted prior to 2010, and the majority were studies within the past 5 years, indicating that the data would reflect the current situation. However, all of the studies reported data collected before the COVID-19 pandemic. COVID-19 pandemic-related confinement has been shown to worsen vitamin D status in several studies outside of Malaysia (61, 62), however it is unclear if the same impact will be observed in Malaysia. We found that individuals living in the urban areas have higher prevalence of vitamin D insufficiency compared to rural areas. This is not surprising considering individuals living in rural areas tend to spend more time outdoor and hence receive higher doses of UVB radiation compared to individuals living in urban areas. Vitamin D deficiency was still detected in the indigenous population, whose lifestyle would involve more outdoor activities and sun exposure. However, the proportion is much lower than the general Malaysian population (51). It should be noted that there was substantial heterogeneity between the studies, which could partly be explained by age group, living in urban areas, gender, and ethnicity. Pregnant women had a much higher risk of vitamin D deficiency than all other age groups for all cut-offs. A longitudinal study followed 179 pregnant women from early pregnancy to birth found that at early pregnancy, 89% had levels <50 nmol/L, and this rose to 96.1% at birth (57). Although 11.1% took vitamin D supplements, none of the women had sufficient vitamin D status at birth.

Our study indicates that vitamin D deficiency/insufficiency in Malaysia can be considered very high compared to other regions worldwide. Table 2 is adapted and updated from a commentary on reviews or systematic reviews of vitamin D status for a country or continent by Bouillon (24). Vitamin D levels < 30 nmol/L in Malaysia are approximately equal to the African continent, but the proportion of levels <50 nmol/L is much higher. However, the prevalence for both cut-offs is comparable to estimates from Mainland China (36). It is also interesting to note that Malaysian Chinese have a much lower proportion of vitamin D insufficiency (35%, <50 nmol/L) compared to Chinese from Mainland China. We are unaware of any studies exploring the difference between Chinese in different international geographical regions and their vitamin D status.

**TABLE 2:** Vitamin D deficiency by geographical area and 25-hydroxyvitamin D cut-offs, updated from Bouillon (24).

	Geographical area	Prevalence, %	
		
		Cut-off of <25 or <30 nmol/L	Cut-off of <50 nmol/L
Hilger et al. (30)	Global	7%	37%
Herrick et al. (31)	USA	5%	18%
Cashman et al. (32)	EU countries (adults)	13%	40%
Arabi et al. (33)	Iran and Jordan	50%	90%
Durazo-Arvizu et al. (34)	Ghana and Seychelles	<1%	<7%
Mogire et al. (35)	African continent	18%	34%
Liu et al. (36)	Mainland China	20.7% (Adults) 23.0% (Adolescents)	63.2% (Adults) 46.8% (Adolescents)
This study	Malaysia	21%	64%

Cut-offs refer to serum concentrations of 25-hydroxyvitamin D used to define vitamin D deficiency.

The prevalence of vitamin D deficiency could be associated to the prevalence of several non-communicable diseases in Malaysia. It has been shown that obese individuals have lower vitamin D levels compared to non-obese subjects (63) due to the reduced bioavailability of the fat-soluble vitamin D (64). According to Malaysia’s National Health and Morbidity Survey 2019, approximately half of Malaysians were above the ideal body weight, with 30.4% overweight and 19.7% obese (65). Vitamin D deficiency has also been linked to the onset of insulin resistance and diabetes mellitus (66). The prevalence of diabetes mellitus in Malaysia is increasing at an alarming rate, with the overall prevalence exceeding 18% in 2019 (67). In addition, the prevalence of raised blood glucose in individuals with unknown diabetes is even higher at 43.4% (95% CI 37.37–49.65) among people aged 65–69 years, which can be attributed to a high intake of sugary beverages (65). In mechanistic animal studies, chronic consumption of high fructose diets can reduce circulating 1,25-(OH)_2_D_3_ (68, 69). In this study, only two articles explored the association between vitamin D status and obesity in our study, but the results were conflicting (70, 71). This prompts for more studies exploring the association of vitamin D and metabolic syndrome in Malaysia.

Dietary vitamin D can be obtained by consuming food that naturally contains vitamin D, fortified food, or supplements. However, limited food choices naturally contain vitamin D in the Malaysian diet (72). Furthermore, vitamin D fortification in Malaysia is voluntary by the manufacturers, with only a few fortifying milks for children and adults (72). The Malaysian Adult Nutrition survey reported that the highest consumer of full cream milk is amongst adults aged 50–59, with only 24% reported daily consumption, while only 15% of individuals aged 18 and 19 reported daily consumption (73). In this meta-analysis, only approximately 10–20% of the participants take vitamin D supplements (57, 60, 74, 75), although the frequency and dosage of supplementation are unclear.

Without significant dietary sources, Malaysians are left to sunlight exposure to achieve adequate vitamin D levels. However, there are numerous reasons why Malaysians tend to avoid sun exposure. Some of the barriers to sun exposure identified amongst Malay women with low vitamin D status include misunderstanding about vitamin D, health concerns toward sun exposure, including effects on skin color and surface, weather (hot and rainy), and religious/cultural clothing practices (76). Additionally, there are limited studies investigating vitamin D synthesis following sun exposure in Malaysia (77). Due to the scarcity of studies, it is unclear exactly how long and how much skin surface is required to be exposed to the sun to obtain adequate vitamin D levels in Malaysia. Different ethnicities further complicate recommendations with different skin tones and different cultural/religious clothing. Hence, more research on sun exposure in Malaysia to meet adequate vitamin D levels is required to provide more explicit guidelines.

There are several notable limitations in this study. Most studies do not report vitamin D supplementation, although some include this information (78). However, vitamin D supplementation is not a common practice among the Malaysian population, and therefore it is unlikely to affect our results significantly. Secondly, 25(OH)D levels were measured using different chromatographic and immunoassays. This is a common issue when comparing studies that involve measurements of vitamin D plasma levels as there is no worldwide standardization (79, 80). Nevertheless, in Mogire’s (35) study, different vitamin D assays accounted for only 5% of the heterogeneity and had no significant impact on the overall 25(OH)D mean concentrations estimate. Thirdly, most of the studies included in the meta-analysis used convenient sampling, which is prone to bias. Finally, although we tried to be inclusive by having a broad inclusion and minimal exclusion criteria, there were limited data for many states in Malaysia.

## Conclusion

Malaysia has a high prevalence of vitamin D deficiency and insufficiency, with more than half of the population estimated to have levels <50 nmol/L. Vitamin D deficiency is more prevalent in Malay and Malaysian Indian ethnic groups than in Malaysian Chinese. The female gender has lower vitamin D levels compared to the male. We strongly recommend immediate public health measures such as the refinement of nutritional guidelines, development of government policies, and awareness campaigns to improve vitamin D status in Malaysia.

## Data availability statement

The requests to access the datasets analysed in this study should be directed to SM, shamin@ukm.edu.my.

## Author contributions

SM, NJ, and NM conducted the literature search. EH, NJ, and SM critically appraised the included studies. SM performed the data analysis and EH and NJ checked it. SM wrote the first draft of the manuscript. All authors contributed to the study’s conception and design and data extraction, commented on previous versions of the manuscript, read, and approved the final manuscript.
