# Supplementary material for: Vitamin D insufficiency is high in Malaysia: A systematic review and meta-analysis of studies on vitamin D status in Malaysia
Source: Front Nutr. 2022 Nov 18;9:1050745. doi: 10.3389/fnut.2022.1050745 (PMC9715981; doi:10.3389/fnut.2022.1050745)
Supplement: Supplementary file 1 [file Data_Sheet_1.docx]

**Supplementary Material 1** Electronic database Search Strategy from inception up to June 2022

| **Database** | **Terms used** | **Hits** |
| --- | --- | --- |
| PubMed | ((((((Vitamin D[MeSH Terms]) OR (Vitamin D Deficiency[MeSH Terms])) OR (Cholecalciferol[MeSH Terms])) OR (Ergocalciferols[MeSH Terms])) OR (Calcifediol[MeSH Terms])) OR (25-Hydroxyvitamin D 2[MeSH Terms])) **AND** ((Malaysia[MeSH Terms]) OR (Malaysia[Text Word])) | 75 |
| Scopus | (TITLE ( "Vitamin D" OR "Vitamin D Deficiency" OR "Cholecalciferol" OR "Ergocalciferols" OR "Calcifediol" OR "25-Hydroxyvitamin D 2" )  **AND** TITLE-ABS-KEY ( Malaysia OR Perlis OR Kedah OR Perak OR Selangor OR "Kuala Lumpur" OR Putrajaya OR Melaka OR "Negeri Sembilan" OR Johor OR Pahang OR Kelantan OR Terengganu OR Sabah OR Sarawak OR Labuan ) ) | 85 |
| Web of Science (WoS) | ts = (" vitamin D" **AND** "Malaysia") | 107 |
| MyJurnal [www.myjurnal.my](http://www.myjurnal.my) | Vitamin D only | 32 |


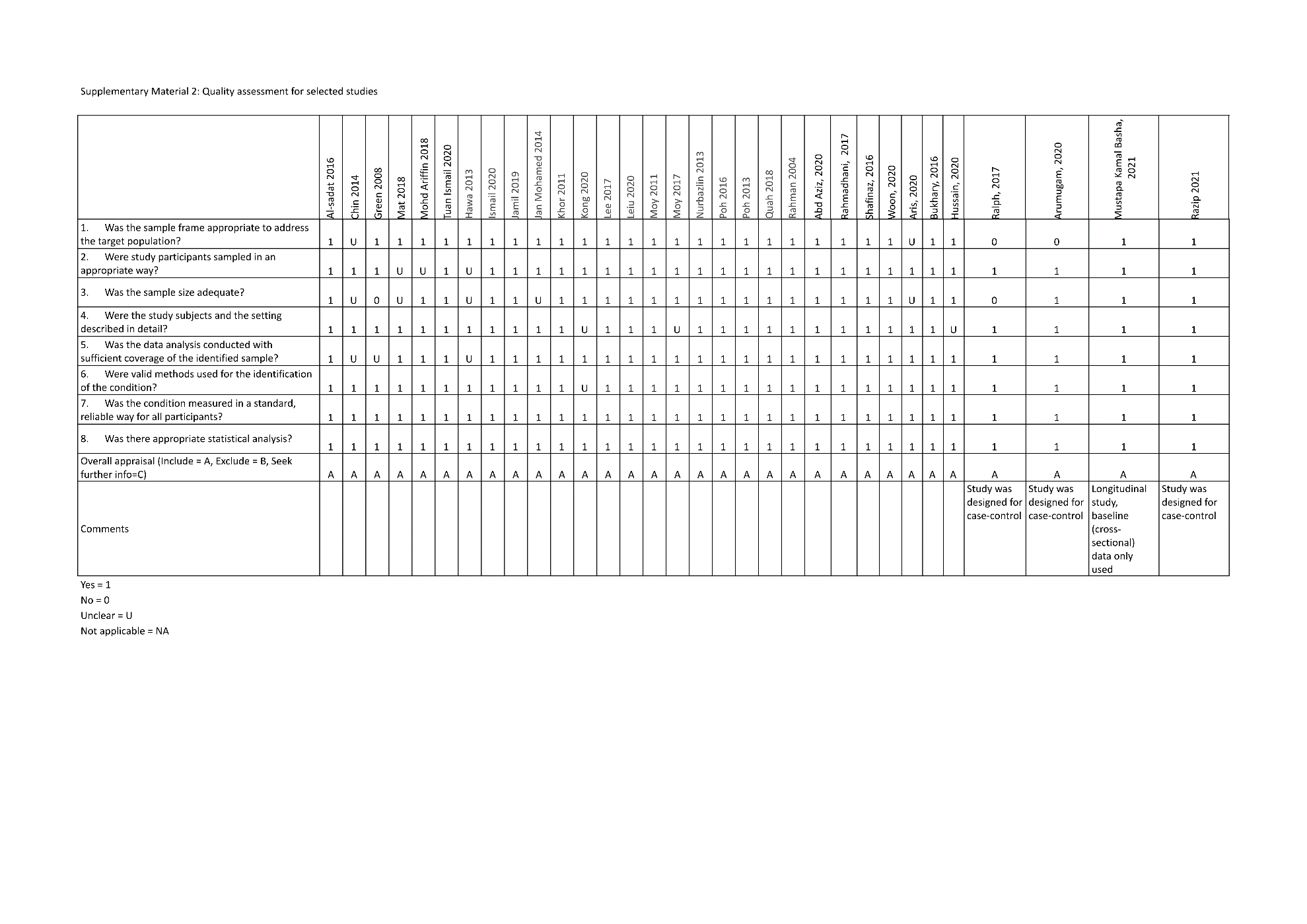

**Supplementary Material 3.** Pooled proportion of 25-hydroxyvitamin D level <50 nmol/L for urban individuals

**Supplementary Material 4.** Pooled proportion of 25-hydroxyvitamin D level <50 nmol/L for rural individuals

**Supplementary Material 5.** Pooled proportion of 25-hydroxyvitamin D level <50 nmol/L for females

**Supplementary Material 6.** Pooled proportion of 25-hydroxyvitamin D level <50 nmol/L for males

**Supplementary Material 7.** Pooled proportion of 25-hydroxyvitamin D level <50 nmol/L for Malay

**Supplementary Material 8.** Pooled proportion of 25-hydroxyvitamin D level <50 nmol/L for Malaysian Indian

**Supplementary Material 9.** Pooled proportion of 25-hydroxyvitamin D level <50 nmol/L for Malaysian Chinese
